# Micelles Mediated Zone Fluidics Method for Hydrazine Determination in Environmental Samples

**DOI:** 10.3390/molecules25010174

**Published:** 2019-12-31

**Authors:** Theano D. Karakosta, Christophoros Christophoridis, Konstantinos Fytianos, Paraskevas D. Tzanavaras

**Affiliations:** 1 LifeLabs Medical Laboratories, Toronto, ON M9W6J6, Canada; karakostatheano@hotmail.com; 2 Environmental Pollution Control Laboratory, Department of Chemistry, Aristotle University of Thessaloniki, 54124 Thessaloniki, Greece; cchrist@chem.auth.gr (C.C.); fyti@chem.auth.gr (K.F.); 3 Laboratory of Analytical Chemistry, Department of Chemistry, Aristotle University of Thessaloniki, 54124 Thessaloniki, Greece

**Keywords:** zone fluidics, hydrazine, *p*-dimethylamino benzaldehyde, micellar medium, water samples

## Abstract

An automated flow method for the determination of hydrazine based on the concept of zone-fluidics has been developed. The analyte reacts under flow conditions with *p*-dimethylamino benzaldehyde (25 mmol L^−1^) in micellar medium (100 mmol L^−1^ SDS) to form a stable derivative (460 nm). Micelles mediated catalysis excludes the use of highly acidic environment typical for this kind of reaction. Following careful examination of chemical and instrumental variables, the method allows the determination of hydrazine at the low micromolar level (0.3–10 μmol L^−1^) in water samples. Real sample analyses (drinking and boiler feed water) resulted in satisfactory results in terms of accuracy with the percent recoveries being in the range of 82–114%.

## 1. Introduction

Hydrazine is a basic compound with interesting chemical properties and high reducing activity. It is being widely used in numerous industrial applications ranging from the production of chemical blowing agents and the synthesis of pharmaceuticals (as intermediate) to polymer science and high-energy rocket propellants [1]. For many years, one of the main uses of hydrazine included high-pressure boilers as an oxygen scavenger. It is oxidized by oxygen to produce nitrogen, passivating oxidized steel, and copper surfaces. One of its main advantages over sulfite, for example, is that potential contamination of the boiler with hydrazine leads to the formation of ammonia, while no dissolved solids are added [2]. Regarding the potential carcinogenic and toxic effects of hydrazine, a recent two-year long systematic study using drinking water in mice and rats concluded that the analyte is carcinogenic in both species, causing statistically significant increase in the incidenses of hepatocellular adenomas and carcinomas [3].

From an analytical point of view, hydrazine can be determined by both spectroscopic and chromatographic techniques [4,5]. Table S1 (Supplementary Material) presents the principles of operation, main analytical figures of merit, and applications of flow methods reporting the determination of hydrazine during the past 15 years [6,7,8,9,10,11,12,13,14,15,16,17,18,19]. One of the most reliable “chemistries” for the analysis of hydrazine in environmental samples is based on its reaction with p-dimethylamino benzaldehyde (*p*-DAB) to form a coloured compound with p-quinone structure. Since its introduction [20], the hydrazine-*p*-DAB reaction has undergone several modifications and improvements [12,21,22] and even been commercialized [23]. The main advantage of the *p*-DAB based procedures is the selectivity since it not based—in contrast to numerous reported assays—on the basis and/or reducing properties of the analyte. On the other hand, a major disadvantage in terms of waste generation and reaction kinetics is the prerequisite for highly acidic medium in order to solubilize the reagent and stabilize the reaction product.

The alternative use of micellar media has proven to be quite effective in acid catalyzed reactions offering enhanced reaction rates and “greener” chemical protocols [24,25]. In brief, above a concentration level that is characteristic for each surfactant (critical micelle concentration), the molecules tend to self-aggregate pointing out the polar groups, while the hydrophobic ones are orientated towards the center [26]. From an analytical point of view, poorly soluble reagents can be easily solubilized in micellar media, fluorescent signals can be enhanced [27], they can serve as additives in chromatographic mobile phases, or even be applied to sample treatment schemes (e.g., cloud point extraction [28]). Analytical applications of micelles can be found in both batch and flow configurations [29,30].

The goal of the present study is to develop a “green” flow method for the selective and sensitive determination of hydrazine in environmental samples at the micromolar level. On this basis, we investigated the potential of replacing the high acidic medium that is typically used in the advantageous and reliable *p*-DAB-hydrazine chemistry with sodium dodecyl sulfate (SDS) and study its behavior under zone-fluidics conditions [31]. The concept of zone fluidics (ZF) is ideal for this purpose as it combines fully automated configurations with reagent and sample consumptions at the microliter levels further enhancing the green character of the whole analytical cycle [32].

## 2. Results and Discussion

### 2.1. Development of the ZF Method

#### 2.1.1. Preliminary Studies

Preliminary studies confirmed that highly acidic medium is necessary for the development of the reaction of *p*-DAB with hydrazine and for the stabilization of the formed derivative. In zone fluidic based-experiments, the optimal concentration range for HCl was found to be 1–2 mol L^−1^ using 50 mmol L^−1^ *p*-DAB.

A second series of preliminary experiments was carried out in order to investigate the possibility of replacing HCl with an alternative micellar medium. Based on the pioneer work of Martinek et al. on the effect of micelles on acid catalyzed reactions [33], we selected the anionic surfactant SDS since it has proved to increase the rate of the reactions between arylamines and arylaldehydes [34]. Indeed, the experiments showed that SDS at the millimolar level can effectively replace HCl for both the preparation of the *p*-DAB solution and the reaction rate and stabilization of the hydrazine–*p*-DAB derivative. It was also shown that although diluted HCl is claimed to favor the micellar reaction in the batch mode [31], there was no practical effect under flow conditions. On the basis of these findings, HCl was completely excluded from subsequent experiments during the development of the flow method.

The investigation of the parameters that potentially affect the performance of the flow method included the reaction time and temperature, the concentration of *p*-DAB and SDS, and instrumental variables such as the volumes of the aspirated zones. The starting values were: V (sample) = 100 μL, V(*p*-DAB) = 50 μL, [SDS] = 100 mmol L^−1^, [*p*-DAB] = 50 mmol L^−1^, Q = 0.4 mL min^−1^, RC = 100 cm, T = RT (25 °C). A standard solution of 5 μmol L^−1^ hydrazine was used in all cases unless otherwise stated.

#### 2.1.2. Effect of Temperature and Time

The reaction temperature proved to have no practical effect on the reaction development. Thermostating the reaction coil at 50 °C using an HPLC column heater (Jones Chromatography) resulted in a less than 5% increase compared to room temperature. No temperature control was therefore adopted for subsequent studies.

**Figure 1:**
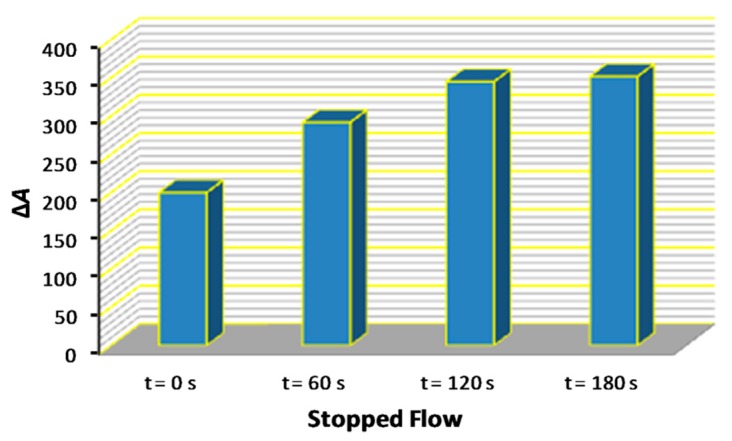
Effect of the reaction time on the sensitivity of the method under stopped-flow conditions.

The effect of the reaction time was investigated by the stopped-flow approach. In brief, following aspiration in the holding coil, the zones were directed through the suitable port towards the 100-cm long reaction coil. Then the flow was stopped, and the mixture was left to react for a specified period of time. The experimental results are shown graphically in Figure 1. The signals practically doubled in the range of 0–120 s and levelled-off thereafter. The stopped-flow reaction time of 120 s was selected for further experiments.

#### 2.1.3. Effect of the Amount Concentrations of *p*-DAB and SDS

**Figure 2:**
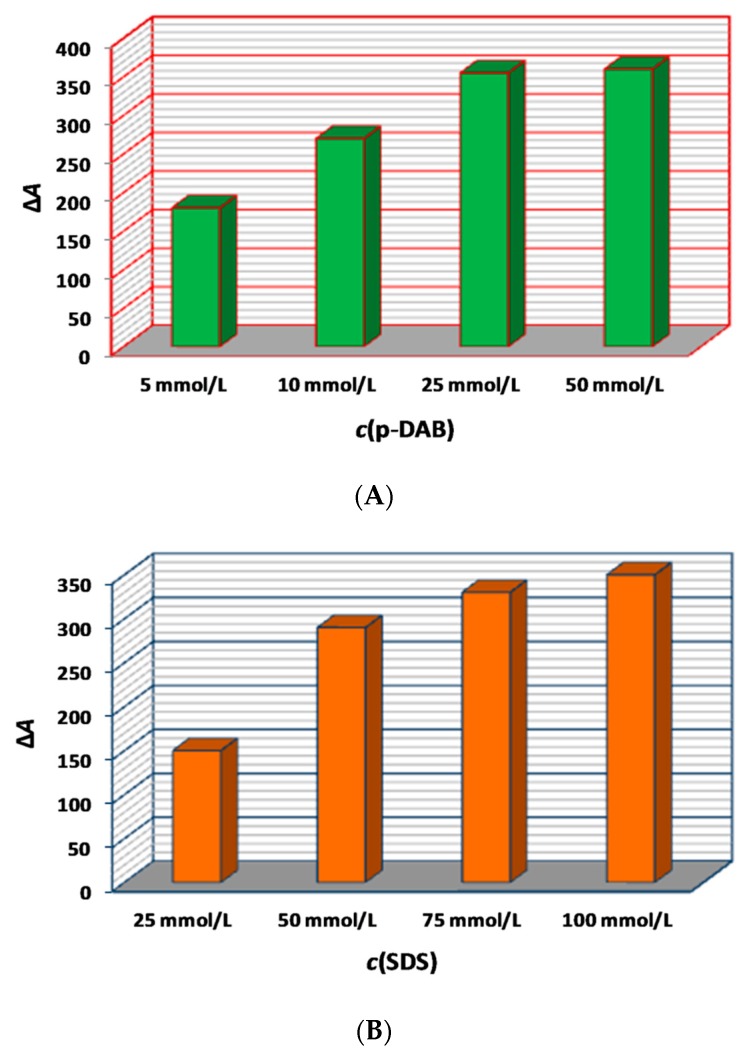
(**A**) Effect of the amount concentration of *p*-DAB. (**B**) Effect of the amount concentration of SDS.

The results from this series of experiments are summarized in Figure 2A,B. Based on the depicted findings, an amount concentration of *p*-DAB of 25 mmol L^−1^ was selected since higher values increased the cost of the method without obvious gain in sensitivity. The amount concentration of SDS had a marking effect in the range of 25–75 mmol L^−1^, resulting in a ca. 2-fold increase in sensitivity (Figure 2B). The value of 100 mmol L^−1^ was selected as optimal.

#### 2.1.4. Effect of Sample and Reagent Volumes

The effect of the sample aspiration volume was almost linear in the range of 50–150 μL, resulting in a 2.5-fold increase in sensitivity due to the minimization of dispersion. The signals practically leveled-off thereafter and the 150 μL volume was selected. On the other hand, the volume of the micelle mediated *p*-DAB reagent had an almost negligible effect on the performance of the method, indicating sufficient excess of the reagent. The initially selected volume of 50 μL was kept as optimal.

### 2.2. Method Validation/Analytical Figures of Merit

Validation of the proposed automated hydrazine method included linearity, limits of detection (LOD), and quantification (LOQ), within and between-day precision.

The linearity of the developed ZF method was evaluated using aqueous solutions of hydrazine (*n* = 8). The experiments confirmed linear behavior in the range of 0.3–10.0 μmol L^−1^, described by the following regression equation (also see Figure S1 in Supplementary section):

*A* = 85.6 (±0.6) [hydrazine] + 10.1 (±2.9) (1)

where A is the absorbance measured as peak height by the flow through photometric detector (mV) and [hydrazine] is the amount concentration of the analyte in μmol L^−1^. The regression coefficient (r) was 0.9996 and the relative error for back-calculated concentrations (residuals) ranged between −1.4 and +0.7% and were distributed randomly along the “zero” axis.

The limits of detection and quantification were estimated using the following equation:

LOD = 3.3 × SD_b_/*a* and LOQ = 10 × SD_b_/*a* (2)

where SD_b_ is the standard deviation of the intercept and *a* is the slope of the regression line [35]. The LOD was therefore calculated to be 0.1 μmol L^−1^ and the LOQ 0.3 μmol L^−1^ hydrazine.

The within-day precision was validated by consecutive injections of standard solutions of hydrazine at 1.0 and 10.0 μmol L^-1^ levels (*n* = 12). The relative standard deviation (RSD) was 1.0% and 0.8%, respectively (see Figure S2 in supplementary section). The between-day precision of the proposed ZF method was evaluated by eight (*n* = 8) non-consecutive calibration curves for a period of 15 days [36]. The RSD of the regression slopes was 4.1%, validating the satisfactory reproducibility of the analytical procedure under the optimal conditions.

### 2.3. Interferences and Matrix Effect

Various species were examined at a 500:1 ratio versus hydrazine such as Cl^−^, CO_3_^2−^, PO_4_^3−^, SO_4_^2−^, Ca(II), Mg(II), EDTA, Pb(II), Cr(III), NH^4+^ and were found not to interfere. Cu(II), Fe(II), Zn(II), Ba(II), and NO^3−^ were tolerated at a 100:1 ratio. Potentially interfering Fe(III) can be masked by either fluorides or reduced using hydroxylamine at a tolerated ratio of 70:1. In all cases, the criterion for interference was set at ±5% variation at a level of 5 μmol L^−1^ hydrazine.

Additional experiments were carried out in order to assess the potential matrix effect that is critical for the applicability of the procedure in real samples. The matrix-effect can be unpredictable, and it may be observed either as positive or negative compared to the response obtained using aqueous standards of hydrazine. The matrix effect was evaluated over the whole concentration range of the analyte by comparing the slopes of the aqueous calibration curve (0.3–10 μmol L^−1^) with matrix-matched calibration curves constructed in individual drinking (tap, mineral, and table) and boiler feed water samples and calculating the relative error between them. As can be seen in Table 1, the matrix effect was less than ±7.6%, being acceptable for the analysis of hydrazine at the micromolar level. Subsequent quantification experiments were therefore carried out using the external aqueous calibration curve in all sample cases.

### 2.4. Analysis of Samples

Various water samples including drinking (tap, mineral, and table) and boiler feed water were treated as described in the Section 3.4. Due to the low stability of the analyte samples were processed with the minimum possible delay and were analyzed in less than 12 h after collection. UPLC coupled to fluorescence detection was used for corroborative purposes based on pre-column derivatization of hydrazine with OPA (see Section 3.5 for experimental details). The results are summarized in Table 2. The presence of hydrazine was confirmed in the samples obtained from boilers at levels in the range of 2.0–3.0 μmo L^−1^ and were in good agreement compared to the UPLC-FL procedure.

Table 3 contains results from the evaluation of the accuracy of the method that was validated by spiking with known amounts of the analyte at 1–2–5 μmol L^−1^. The percent recoveries ranged between 82% and 114%.

## 3. Materials and Methods

### 3.1. Instrumentation

All experiments were performed using a ZF configuration made in-house that comprised the following parts (Figure S3, supplementary data): (i) A low pressure micro-electrically actuated 10-port valve (Valco, Thessaloniki, Greece), (ii) Minipuls3 (Gilson, Middleton, WI, USA) peristaltic pump, (iii) a reaction coil heater (Jones Chromatography), and (iv) a flow through photometric detector (FIAStar 5023, Foss-Tecator, Hilleroed, Denmark). The holding coil was 300 cm × 0.7 mm i.d., while the reaction coil (100 cm × 0.5 mm i.d.) was knitted tightly around a 4.6 mm i.d. stainless-steel rod allowing efficient heating when applicable. A special program based on LabVIEW 5.1.1 instrumentation software package (National Instrument, Austin, TX, USA) was used for the operation and control of the ZF setup, while the Clarity^®^ software (DataApex, Prague, Czech Republic) was selected for peak-height acquisition.

### 3.2. Reagents and Solutions

All reagents were of analytical grade and purchased by either Merck (Darmstadt, Germany) or Sigma (St. Louis, MO, USA) unless stated otherwise. Purified water was produced by a Milli-Q system (Millipore, St. Louis, MO, USA). The standard stock solution of hydrazine was prepared daily in 0.01 mol L^−1^ HCl at an amount concentration of 10 mmol L^−1^. Working solutions at the micromolar level were prepared by serial dilutions in the same solvent. A reagent solution of p-dimethylamino-benzaldehyde (*p*-DAB, c = 25 mmol L^−1^) was prepared daily by dissolving the reagent in 100 mmol L^−1^ sodium dodecyl sulphate (SDS). All other solutions used for selectivity studies were prepared in water from analysis grade sodium/potassium or chloride/sulphate salts for anions and cations, respectively.

### 3.3. Analytical Procedure

**Figure 3:**
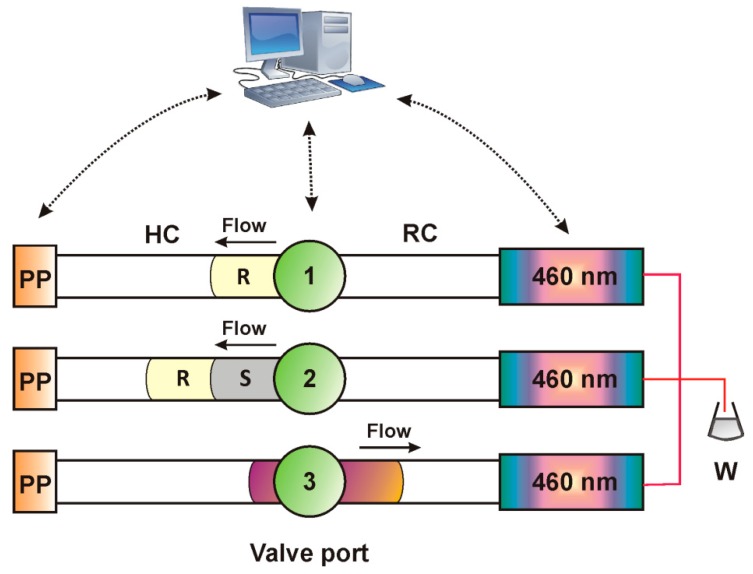
Graphical depiction of the zone fluidics analytical sequence steps for the determination of hydrazine; PP = peristaltic pump, HC = holding coil, S = sample, R = *p*-DAB/SDS reagent, RC = reaction coil, W = waste.

A simple two-zone approach was selected for the determination of Hydrazine. As can be seen graphically in Figure 3, zones of the micellar mediated *p*-DAB reagent (R = 50 μL) and the sample (S = 150 μL) were aspirated in the holding coil of the ZF system (Step 1, S1). The second step (S2) involved propulsion of the mixture at a flow rate of 0.4 mL min^−1^ towards the 100-cm knotted reaction coil and reaction development for 120 s. During Step 3 (S3), the product was detected on passage through the flow cell of the photometric detector (460 nm). Signals were evaluated based on peak height, while three replicates were made in all instances. The practical sampling rate was 15 h^−1^.

### 3.4. Preparation of Samples

Several drinking and boiler feed water samples were analyzed in order to evaluate the applicability of the developed method. All samples were collected in airtight containers avoiding agitation and exposure to the air [23]. Tap water samples were collected from different locations of the city of Thessaloniki, while bottled table and mineral water samples were commercially available. Boiler feed water samples were kindly provided by local companies. In all cases, the samples were processed either immediately or with the least possible delay (<12 h from collection). Sample treatment involved filtration (when necessary) and acidification by adding 1 mL of 1 mol L^−1^ HCl to 99 mL of each sample.

### 3.5. UPLC-FL Corroborative Method

An in-house developed and validated UPLC-FL method has been utilized for corroborative purposes. The method is based on pre-column derivatization of hydrazine with o-phthalaldehyde (OPA) in acidic pH followed by UPLC reversed phase separation and fluorimetric detection.

#### 3.5.1. Instrumentation and Reagents

An Acquity UPLC binary solvent system (Waters) equipped with a fluorescence detector was employed. The analytical column was a reversed phase Acquity UPLC C18 BEH (50 × 2.1 mm, 1.7 μm, Waters, Milford, MA, USA). The data acquisition and the instrument control were carried out via the Empower 2 Pro software.

The OPA derivatization reagent (25 mmol L^−1^) was prepared by dissolving the reagent in 0.5 mL of MeOH followed by dilution with 9.5 mL water. A 100 mmol L^−1^ phosphate buffer was prepared and the pH was adjusted to 2 by dropwise adding of concentrated H_3_PO_4_.

#### 3.5.2. Derivatization and Chromatography

All separations were made using a binary gradient elution program. The mobile phases A and B were a 20 mmol L^−1^ phosphate buffer (pH = 2) and acetonitrile, respectively. The initial ratio was 2% *v*/*v* of B and kept constant for 1 min and then linearly increased to 10% in 1 to 2 min and then change to the initial composition (2% B) in 2 to 5 min followed by a column equilibrium period of 5 min to obtain reproducible separations. The flow rate was set to 0.50 mL min^−1^ while the injection volume was 5 μL. The column was thermostated to 30 °C. The hydrazine-OPA derivative was detected spectrofluorimetrically at λ_ex_/λ_em_ = 315/370 nm. Between injections the autosampler was sequentially washed with 1000 μL of water/CH3OH (90/10 *v*/*v*) and water/CH3OH (10/90 *v*/*v*) to remove any sample residuals.

A volume of 700 μL hydrazine standard or sample were vortex mixed for 10 s with 100 μL OPA and 200 μL phosphate buffer in a suitable vial. The mixture was allowed to react for 5 min at ambient temperature and 5 μL were injected in the UPLC-FL system for subsequent separation and analysis.

## 4. Conclusions

The proposed method offers some interesting features that can be summarized as follows:

(i) The reaction of *p*-DAB with hydrazine has been automated under zone fluidics conditions offering flow automation with minimum reagents consumption and waste generation.
(ii) The highly acidic environment typical for *p*-DAB reactions has efficiently been replaced with micellar medium (SDS) with equal performance and figures of merit.
(iii) The developed method offers adequate sensitivity and selectivity for the determination of hydrazine at the low micromolar level and an analysis throughput of 15 h^−1^.
(iv) The results were validated by comparison to a UPLC-FL method based on pre-column derivatization with OPA.

## Figures and Tables

**Table 1:** Evaluation of the matrix effect.

Calibration Curve	Slope	SD	Matrix Effect (%) ^a^
Aqueous	85.6	0.6	–
Tap Water 1	81.1	0.8	−5.3
Tap Water 2	81.6	0.8	−4.7
Mineral Water 1	88.9	1.1	+3.9
Mineral Water 2	89.9	1.0	+5.0
Table Water 1	89.5	1.1	+4.6
Table Water 2	80.6	0.9	−5.8
Boiler water 1	91.9	1.6	+7.4
Boiler water 2	79.1	1.3	−7.6

^a^ The matrix effect was calculated by the ratios of slopes of the matrix-matched (individual samples) to the aqueous calibration curve.

**Table 2:** Analysis of real samples by the zone fluidics (ZF) method.

Sample	Hydrazine Found (μmol L^−1^)	UPLC-FL
Boiler water 1	2.1	1.9
Boiler water 2	2.8	2.5
Tap Water 1	N.D.	N.D.
Tap Water 2	N.D.	N.D.
Mineral Water 1	N.D.	N.D.
Mineral Water 2	N.D.	N.D.
Table Water 1	N.D.	N.D.
Table Water 2	N.D.	N.D.

N.D. = not detected.

**Table 3:** Accuracy of the ZF method.

Sample	Hydrazine Added (μmol L^−1^)	% Recovery ZF	% Recovery UPLC-FL
Boiler water 1	1.0	86	92
	2.0	88	102
	5.0	92	95
Boiler water 2	1.0	82	94
	2.0	110	89
	5.0	99	106
Tap Water 1	1.0	114	86
	2.0	91	89
	5.0	92	89
Tap Water 2	1.0	108	96
	2.0	96	99
	5.0	110	91
Mineral Water 1	1.0	89	92
	2.0	86	109
	5.0	87	106
Mineral Water 2	1.0	102	108
	2.0	95	110
	5.0	112	103
Table Water 1	1.0	111	91
	2.0	84	84
	5.0	85	113
Table Water 2	1.0	109	91
	2.0	113	90
	5.0	95	108

## Supplementary Materials

The following are available online, Figure S1: Graphical depiction of the aqueous calibration curve, Figure S2: Representative ZF peaks, Figure S3: Schematic depiction and image of the ZF configuration (for experimental details on the instrumentation see the experimental Section 3.2), Table S1: Overview of automated flow methods for the determination of hydrazine.
