# Supplementary material for: Micelles Mediated Zone Fluidics Method for Hydrazine Determination in Environmental Samples
Source: Molecules. 2019 Dec 31;25(1):174. doi: 10.3390/molecules25010174 (PMC6983152; doi:10.3390/molecules25010174)
Supplement: Supplementary file 1 [file molecules-25-00174-s001.pdf]

Article

Micelles Mediated Zone Fluidics Method for Hydrazine Determination in Environmental Samples

Theano D. Karakosta [1], Christophoros Christophoridis [2], Konstantinos Fytianos [2] and Paraskevas D. Tzanavaras [3,*]

[1] LifeLabs Medical Laboratories, Toronto, ON M9W6J6, Canada; karakostatheano@hotmail.com
[2] Environmental Pollution Control Laboratory, Department of Chemistry, Aristotle University of Thessaloniki, 54124 Thessaloniki, Greece; cchrist@chem.auth.gr (C.C); fyti@chem.auth.gr (K.F.)
[3] Laboratory of Analytical Chemistry, Department of Chemistry, Aristotle University of Thessaloniki, 54124 Thessaloniki, Greece
* Correspondence: ptzanava@chem.auth.gr; Tel.: +30-2310997721, Fax: +30-2310997719

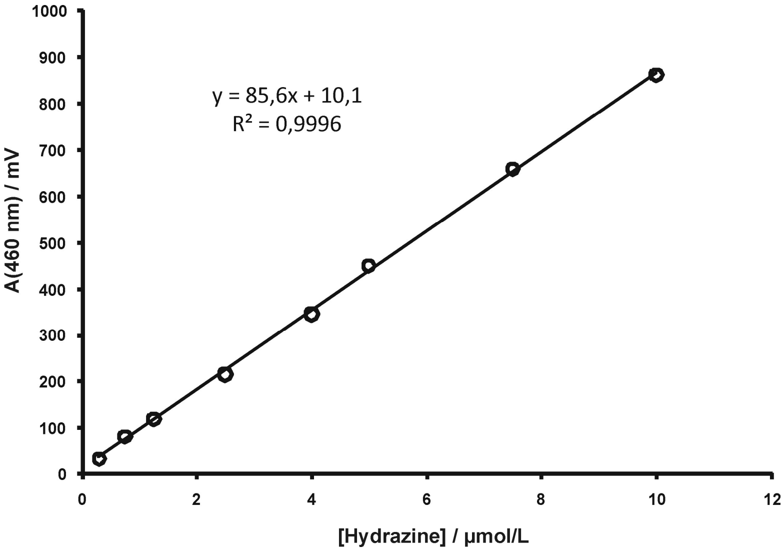

Figure 1S. Graphical depiction of the aqueous calibration curve.

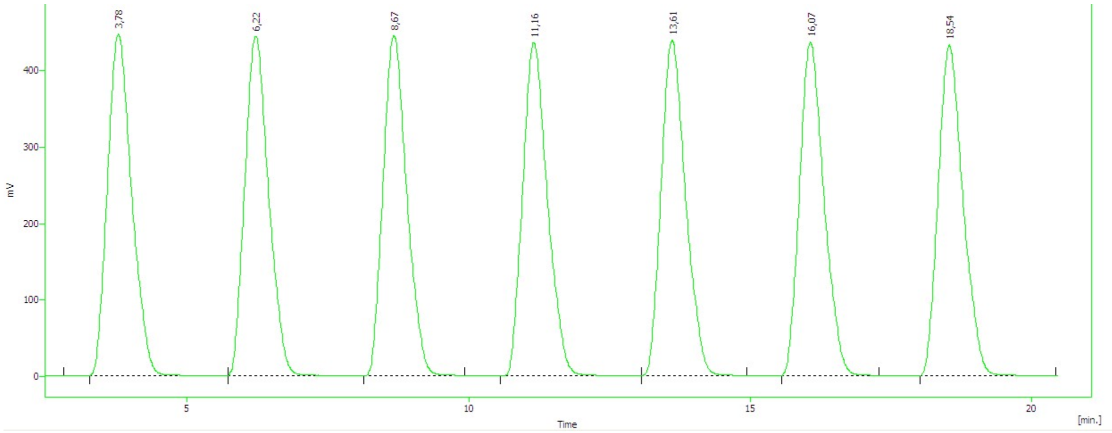

Figure 2S. Representative ZF peaks.

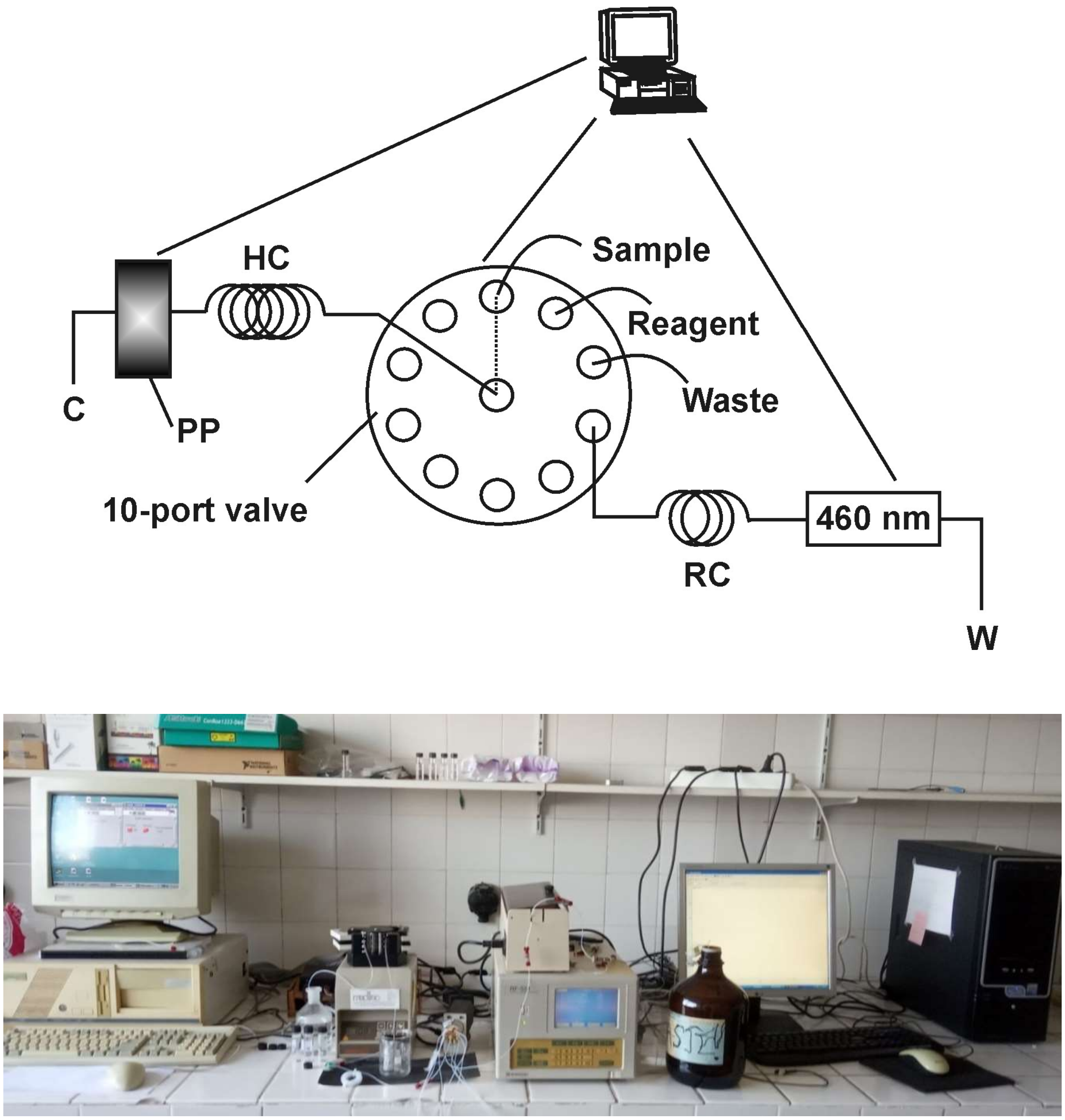

Figure 3S. Schematic depiction and image of the ZF configuration (for experimental details on the instrumentation see the experimental section 3.2).

Table 1S. Overview of automated flow methods for the determination of hydrazine.

Flow Technique	Method principle	LOD (μg L-1)	Sampling Rate (h-1)	Application	Ref
ZF	Reaction with OPA after on-line SPE and fluorimetric detection (318/376 nm)	0.9	12	Pharmaceutics	[6]
FI	Amperometric sensor based on electrocatalytic oxidation at an Au nanoparticle-modified pretreated graphite pencil electrode	0.07	120	Water samples	[7]
FI	Amperometric sensor based on electrocatalytic oxidation at pyrocatechol violet modified pencil graphite electrode	2.9	—	Water samples	[8]
FI	Electrochemical detection with a coplanar boron doped diamond microband electrode modified with Pt nano-particles	2.3	—	Pharmaceutics	[9]
FI	Electrochemical detection with a Quercetin tethered pristine-multiwalled carbon nanotube modified glassy carbon electrode	4.9	10	Cigarette smoke	[10]
FI	Voltammetric detection with a nanostructured $AuCu_3$ alloy electrode	1.4	45	Water samples	[11]
FI	Reaction with p-DAB in highly acidic medium and spectrophotometric detection (460 nm)	2 (LOQ)	24	Water samples	[12]
FI	Amperometric determination using a surface modified nickel hexacyanoferrate graphite electrode	36	54	Water samples	[13]
FI	Indirect AAS using PbO_2 as solid phase microcolumn packing.	101	80	Water samples	[14]
FI	Amperometric detection by cyclic voltammetry using ruthenium modified glassy carbon electrodes	306	—	N/A	[15]
FI	Electrocatalytic determination using copper-palladium alloy nanoparticle plated screen-printed carbon electrodes	9.7	180	Cigarette smoke	[16]
FI	Elelctrocatalytic determination using barrel plating rhodium disposable electrodes	2.5	45	Water samples	[17]
FI	Post-chemiluminescence arising from the permanganate-luminol reaction induced by hydrazine	30	—	N/A	[18]
FI	Inhibition of the pyrogallol red-iodate reaction (470 nm)	30	15	Water samples	[19]
ZF	Reaction with p-DAB in micellar medium and UV-vis detection (460 nm)	3.6	15	Water samples	This study

ZF = zone fluidics; **OPA** = *o*-phthalaldehyde; **SPE** = solid phase extraction; **FI** = flow injection; **N/A** = no application in real samples.
